# Dual maternal origin and dispersal history of peach (*Prunus persica*) from China

**DOI:** 10.1093/aob/mcag248

**Published:** 2026-08-05

**Authors:** Xing Kong, Raees Khan, Zeng-Yuan Wu, Jie Cai, Jie Liu

**Affiliations:** Germplasm Bank of Wild Species & Yunnan Key Laboratory of Crop Wild Relatives Omics, Kunming Institute of Botany, Chinese Academy of Sciences, Kunming 650201, China; University of Chinese Academy of Sciences, Beijing 100049, China; Germplasm Bank of Wild Species & Yunnan Key Laboratory of Crop Wild Relatives Omics, Kunming Institute of Botany, Chinese Academy of Sciences, Kunming 650201, China; Germplasm Bank of Wild Species & Yunnan Key Laboratory of Crop Wild Relatives Omics, Kunming Institute of Botany, Chinese Academy of Sciences, Kunming 650201, China; Germplasm Bank of Wild Species & Yunnan Key Laboratory of Crop Wild Relatives Omics, Kunming Institute of Botany, Chinese Academy of Sciences, Kunming 650201, China; Germplasm Bank of Wild Species & Yunnan Key Laboratory of Crop Wild Relatives Omics, Kunming Institute of Botany, Chinese Academy of Sciences, Kunming 650201, China

**Keywords:** Domestication, maternal origin, peach, phylogeography, population demography, *Prunus persica*

## Abstract

**Background and Aims:**

Compared with annual crops, the domestication of perennial fruit trees remains poorly understood, and tracing the maternal origins and dispersal history significantly advances our understanding of the domestication process. Peach (*Prunus persica*) is a vital economic fruit crop with a long and intricate history of human domestication, resulting in diverse varieties and wide dispersal, making it an excellent model for tracing the maternal origin and domestication history. Here we aim to investigate the peach’s maternal origins and elucidate its domestication history in China.

**Methods:**

We investigated peach phylogeography by analysing 714 peach individuals representing diverse germplasm types across its distribution range, using the whole chloroplast genome and nuclear ribosomal DNA sequences to further infer its maternal origin and domestication history.

**Key Results:**

Genetic structure analyses of both datasets revealed two distinct clades within peach populations (Clade I and Clade II), each associated with similar geographic distributions and containing various germplasm types with diverse fruit phenotypes, suggesting that peach is derived from two distinct maternal ancestors (dual maternal origin). Notably, genetic diversity and private haplotype analysis identified multiple regions with high diversity (the Yellow River, the middle and Yangtze River and the Pearl River basin) where the oldest fossil records are concentrated, suggesting that peach might exist in multiple domestication regions (diffuse origins). Peach populations experienced two major bottlenecks and sequential founder events during the domestication process, likely shaped by human-driven selection and dispersal: the first occurred around 6000 years ago and the second approximately 2000 years ago.

**Conclusions:**

This study highlights a dual maternal origin and multiple domestication regions of peach within China, resolving a key issue in the history of peach domestication. Our integration of chloroplast and nuclear genome data, along with other lines of evidence, establishes a clear framework for inferring complex lineages, refining our understanding of the peach demographic history, and offering valuable insights for breeding strategies and conservation efforts.

## INTRODUCTION

Plant domestication is a significant evolutionary process driven by human selection alongside environmental pressures that has profoundly shaped human civilization over thousands of years, transforming the course of human history (Zohary *et al.*, 2012; Fuller *et al.*, 2023; Gutaker and Purugganan, 2024). Early investigations focused on morphological, physiological and ecological aspects, highlighting the interplay between anthropogenic selection, environmental factors and inherent genetic variability (Zeven, 1973; Ross-Ibarra *et al.*, 2007; Zeder, 2015). Modern genomic technologies have enabled the identification of domestication genes and evolutionary trajectories of major crops (Burke *et al.*, 2007; Gepts, 2014; Mascher *et al.*, 2024). However, significant gaps remain in our understanding of the maternal origins of domesticated plants (a lineage traced through chloroplast DNA), an area essential for reconstructing crop domestication histories and informing effective breeding strategies (Agrawal *et al.*, 2014; Soltis and Soltis, 2021).

The chloroplast genome is characterized by its small size, haploid status, lack of recombination, uniparental inheritance and low mutation rates, making it a powerful tool for tracing maternal lineages and reconstructing plant evolutionary and dispersal histories (Triest *et al.*, 2021; X. Y. Song *et al.*, 2024). It has been widely used to study genetic relationships within and among populations (Jayaswall *et al.*, 2022; Zhang *et al.*, 2023; Xia *et al.*, 2024; Khan *et al.*, 2026) and to elucidate plant phylogeny, biogeography and evolutionary trajectories (Wu *et al.*, 2022; Khan *et al.*, 2026; Fu *et al*., 2026). Chloroplast genomic studies can provide a unique perspective on domestication selection, genetic relationships and adaptation to diverse ecological habitats (Gepts, 2014; Gao *et al.*, 2019). Maternal lineage, mainly inherited through chloroplast genomes, plays a crucial role in shaping the genetic diversity of crops (Nodine and Bartel, 2012; Barnbrook *et al.*, 2023; Okada and Watanabe, 2024). Consequently, comprehensive analyses of the chloroplast genome in domesticated crops are essential to provide a complete picture and will advance our understanding of domestication processes (Haas *et al.*, 2019; Sun *et al.*, 2021; Jenny *et al.*, 2024).

Peach (*Prunus persica*, Rosaceae) is a globally economically significant and widely consumed fruit, primarily cultivated for its edible fruits and ornamental flowers in temperate regions, and is a compelling case study (Abbott *et al.*, 2002; Lu *et al.*, 2003). Peach ranks as the second (following apple) most produced temperate fruit crop, with China as the global leading producer (Manganaris *et al.*, 2022).

A wide consensus indicates that peaches originated in China, where they were domesticated and from where they have since spread to various regions (Das *et al.*, 2011; Zheng *et al.*, 2014; Ezzat *et al.*, 2024; Li *et al.*, 2025), supported by both fossil and archaeological evidence. Fossil evidence, including the presence of peach endocarps, indicates that its wild ancestor existed as early as 2.6 million years ago in south-western China (Su *et al.*, 2023). Archaeological evidence further shows that human use of peaches stretches back to around 7500 years ago in the middle and lower Yangtze River regions (Zheng *et al.*, 2014). Nuclear genome studies support the peach’s origin in south-west China (Yu *et al.*, 2018) and subsequent dispersal westward after domestication, likely facilitated by the historical trade routes, i.e. the Silk Road (which played a key role in the westward expansion of peach from China to Central Asia and beyond), accompanied by a reduction in genetic diversity compared with their wild relatives, likely due to domestication and sequential founder events during dispersal (Verde *et al.*, 2013; Cao *et al.*, 2014, 2022; Wang *et al.*, 2021; Li *et al.*, 2024, 2025). Despite these advances, the origin and domestication history of the peach remain controversial (Yu *et al.*, 2018), and its direct wild ancestor populations are still unknown (Yu *et al.*, 2018). Specifically, the maternal domestication history, including the number of maternal origin regions and the precise dispersal routes, remains poorly understood. In peach evolution, cpDNA markers based on the chloroplast genome have successfully distinguished domesticated peach from its wild congeners (e.g. *P. mira* and *P. davidiana*), identifying unique maternal signatures of the peach lineage (Badenes and Parfitt, 1995). More recently, the use of the chloroplast genome has further resolved well-supported phylogenies across *Prunus* and uncovered complex evolutionary patterns such as cytonuclear discordance (Su *et al.*, 2023). Therefore, with the increasing availability of genomic resources (Yu *et al.*, 2018), it is now possible to assemble a large number of chloroplast genomes, making it feasible to trace the maternal origin of peach using chloroplast genome (plastome) phylogeography (X. Y. Song *et al.*, 2024; Li *et al.*, 2026).

To address these specific gaps in the knowledge about the origin and dispersal of peach, we leveraged published genomic resources alongside our new investigation, analysing 714 peach individuals from around the world. Using whole chloroplast genomes and nuclear ribosomal DNA sequences, we aimed to elucidate the maternal origin and dispersal history of the peach. The specific objectives were to (1) elucidate the phylogeographic structure of peach from a maternal lineage perspective; (2) assess genetic diversity among peach populations from different regions; and (3) infer the maternal origin and domestication regions of cultivated peach. We will address these objectives with a maternal lineage perspective to resolve uncertainties in peach domestication history. The results will provide valuable information for the conservation and management of peach germplasm and serve as a case study for investigating maternal origins in other crop species.

## MATERIALS AND METHODS

### Taxon sampling

A total of 714 peach individuals were used in this study, including 687 individuals from previous studies (Cao *et al.*, 2019, 2022; Li *et al.*, 2019; Guo *et al.*, 2020; Yu *et al.*, 2021; Zhou *et al.*, 2023) and 27 newly sampled individuals (Supplementary Data Fig. S1, Supplementary Data Table S1). These samples were grouped into datasets, with all samples collectively referred to as the (1) AL group. Based on the degree of human influence (Guo *et al.*, 2020; Cao *et al.*, 2022; Zhou *et al.*, 2023), the samples were further classified into the following groups: (2) LW group (Landrace and wild), comprising 182 landrace accessions and 27 wild accessions, representing more traditional populations (landraces are defined here as traditionally cultivated, locally adapted populations likely maintained by seed propagation); (3) CL group (cultivated ornamental and improved cultivars), including 42 ornamental accessions and 405 improved accessions due to their frequent controlled crossbreeding; and (4) NA group, consisting of 58 individuals with unknown germplasm type classification. Additionally, other Rosales species (30 species from 19 genera as outgroups) were included in the phylogenetic analyses (Supplementary Data Table S2). Voucher specimens were collected for each newly sampled individual.

### Data download, DNA extraction, sequencing

SRA Toolkit v3.0.2 (https://github.com/ncbi/sra-tools) was used to download 687 peach resequencing files in Sequence Read Archive (SRA) format from the National Center for Biotechnology Information (NCBI) website (https://www.ncbi.nlm.nih.gov/). The fasterq-dump function from the SRA Toolkit, combined with pigz v2.3 (https://zlib.net/pigz/), was used to retrieve sequencing data in fastq.gz format.

For the 27 newly sampled individuals, total genomic DNA was extracted from ∼0.20 g of clean, young and healthy leaves dried with silica gel using a modiﬁed CTAB method (Doyle and Doyle, 1987). DNA quality and quantity were assessed using 1 % agarose gel electrophoresis and a NanoDrop 2000 spectrophotometer (Thermo Fisher Scientific, Wilmington, DE, USA). High-quality DNA samples were sheared into fragments of ∼200–500 bp using sonication. DNA libraries were constructed using the NEBNext Ultra II DNA Library Prep Kit for Illumina (New England BioLabs, MA, USA), following the manufacturer’s protocol. Whole-genome sequencing was performed on the BGI DNBSEQ-T7 platform by BGI Group (Shenzhen, China), using paired-end reads of 150 bp. Each individual was sequenced to a depth of at least 25×, generating >7 Gb of clean data per sample.

### Chloroplast genome and nrDNA sequence assembly and annotation

Two datasets of peach were constructed for downstream analysis: the complete chloroplast genome (cpDNA) and the nucleotide ribosomal DNA (nrDNA). The sequences were assembled from the resequencing data using GetOrganelle (Jin *et al.*, 2020), with peach (GenBank accession NC014697.1) and *P. mahaleb* (GenBank accession MT796547.1; the identity between them is 99.06 %) serving as reference sequences for cpDNA and nrDNA, respectively. Annotation and verification of the assembled sequences were conducted using Geneious v2022.0.2 (Kearse *et al.*, 2012).

### Haplotype diversity, population structure and haplotype genealogy construction

The MAFFT tool (Katoh and Standley, 2013) was used to align all assembled and annotated chloroplast DNA and nuclear ribosomal DNA sequences and subsequently calculated the number of haplotypes (*H*), haplotype diversity (*H*_d_) and nucleotide diversity (*π*) in different peach groups using DnaSP v6.12.03 (Rozas *et al.*, 2017) with default parameters. Due to different groups, some individuals belonged to private haplotypes in one of these three groups (CL/LW/NA), but it was classified as a shared haplotype in the AL group. Therefore, the AL group, including all individuals, was used to count the haplotype numbers of different groups. To compare the haplotype composition of the AL group and germplasm types, we utilized the R package *ggplot2* (Wickham, 2011) to visualize it.

Maximum likelihood (ML) and Bayesian inference (BI) methods were used to reconstruct the phylogenetic tree based on cpDNA and nrDNA datasets to explore the phylogenetic relationship among haplotypes. The ML tree was generated using the GTR + G + I model of the Akaike information criterion (AIC) as the optimal model of nucleotide substitution in jModelTest v2.1.7 (Posada, 2008), with bootstrap values computed after 1000 replicates by RAxML v8.2.11 (Stamatakis, 2014). MrBayes v3.2.7a (Ronquist *et al.*, 2012) was used to construct the BI tree by performing four Markov chain Monte Carlo (MCMC) simulations and ran for 1 000 000 generations with sampling of trees every 1000 generations, and the first 25 % of the trees were discarded as burn-in; the remaining were used to create the majority-rule consensus tree. As the ML and BI trees exhibited identical topological structures, the ML tree was selected for presentation. Bootstrap support values (>95 %) and Bayesian posterior probabilities (>0.95) were both annotated on the same nodes. The tree was visualized and annotated using FigTree v1.4.4 (http://tree.bio.ed.ac.uk/software/figtree/) and iTOL (Interactive Tree of Life) (http://itol.embl.de/).

We conducted the haplotype network analysis with the TCS method in PopART v1.7 (Leigh *et al.*, 2015), while principal components analysis (PCA) was performed with R package adegenet (Jombart, 2008). To explore the population structure, we used the Bayesian clustering method to conduct a cluster analysis for the LW group in STRUCTURE v2.3.4 (Pritchard *et al.*, 2000) with ten independent simulations run for each *K* (*K* = 1–10) with a burn-in 50 000 steps followed by 100 000 MCMC iterations. Structure Harvester (Earl and VonHoldt, 2012) was applied to select the optimal *K* value. Furthermore, CLUMPP v1.1.2 (Jakobsson and Rosenberg, 2007) was used to generate a consensus *Q* matrix and we then used the R package ggplot2 to create bar plots.

### Genetic diversity and spatial structure

The haplotype diversity (*H*_d_) and nucleotide diversity (*π*) of the LW group based on cpDNA and nrDNA datasets were calculated using DnaSP v6.12.03 (Rozas *et al.*, 2017). To detect genetic differentiation, we used the R package Pheatmap to perform a genetic distance analysis among the LW group haplotypes and Arlequin v3.5 (Excoffier and Lischer, 2010) to assess pairwise genetic differentiation (*F*_ST_) between two different clades in the LW group and three different peach accessions with 1000 permutations. The R package *ggplot2* was used to visualize all results. To analyse the spatial distribution of haplotypes, we mapped haplotype numbers and their proportion of the LW and CL groups in different clades with their geographic distribution using ArcMap v10.7 (ESRI, Redlands, CA, USA).

### Population demography and divergence time estimate

To evaluate the population demographic history for each clade within the LW group Bayesian, a Bayesian skyline plot (BSP) was constructed using BEASTv1.10.4 (Drummond and Rambaut, 2007) to infer detailed characteristics of demographic history according to the effective population size dynamics based on cpDNA. The parameters used were a strict clock model, a coalescent Bayesian skyline model prior, and an MCMC chain length of 20 000 000 in BEAUTi v1.8 (Drummond *et al.*, 2012). The output was checked, making sure that the effective sample size (ESS) value was >200 using Tracer v1.7.2 (Rambaut *et al.*, 2018).

To estimate the divergence time of the two peach clades, we combined haplotypes of the LW group with published chloroplast genomic data of Rosales species (downloaded 28 species from 19 genera belonging to three subfamilies in NCBI as outgroups) (Supplementary Data Table S2). Four calibration points were employed for divergence time estimation: (1) 101.05 Ma for the stem age of Rosales (Wang *et al.*, 2009); (2) 55 Ma for the stem age of Prunoideae/*Prunus* lineage (Li *et al.*, 2011); (3) 48.6 Ma for the stem age of Rosoideae/*Rosa* lineage (Edelman, 1975); (4) 45 Ma for the crown age of Maloideae/*Malus* lineage (Velasco *et al.*, 2010). All these calibration points were performed with normal distribution and a standard deviation of 1–3. BEAST v1.8.0 (Drummond and Rambaut, 2007) was used to run the analysis with a Yule model, an uncorrelated lognormal relaxed clock, and with an MCMC chain length of 800 000 000, with sampling at every 1000 generations. The reliability of the results was assessed by checking that the ESS value was >200 using Tracer v1.7.2 (Rambaut *et al.*, 2018). The MCC tree was generated after discarding the initial 20 % as burn-in using TreeAnnotator v1.10.4 (Drummond and Rambaut, 2007). Finally, FigTree v1.4.4 was used to visualize the MCC tree with mean ages and 95 % highest posterior density (HPD).

## RESULTS

### Population structure based on LW and AL groups

The phylogenetic analyses of both datasets (cpDNA and nrDNA) show congruence, revealing that *P*. *persica* is monophyletic, and four published *P. persica* chloroplast genomes and one *P. ferganensis* genome were included, with *P. ferganensis* nested within *P. persica*, supporting its classification as a synonym (Figs 1B and 2B). The phylogenetic analysis of cpDNA haplotypes and nrDNA ribotypes within the LW group consistently optimally divided into two distinct clades (Clade I and Clade II), and these findings were congruent with principal component analysis (PCA), STRUCTURE analysis (optimal *K* = 2), and haplotype network analysis (Fig. 1C–E and Supplementary Data Fig. S2c, d). The two clades exhibit broadly sympatric geographical distribution across China; however, Clade II displayed a wider geographic distribution range, extending to regions such as the Xinjiang Autonomous Region in China. In addition, the phylogenetic tree based on cpDNA among the AL group confirmed the stable existence of two clades (i.e. Clade I and Clade II) (Supplementary Data Figs S3 and S4).

**Fig. 1.**
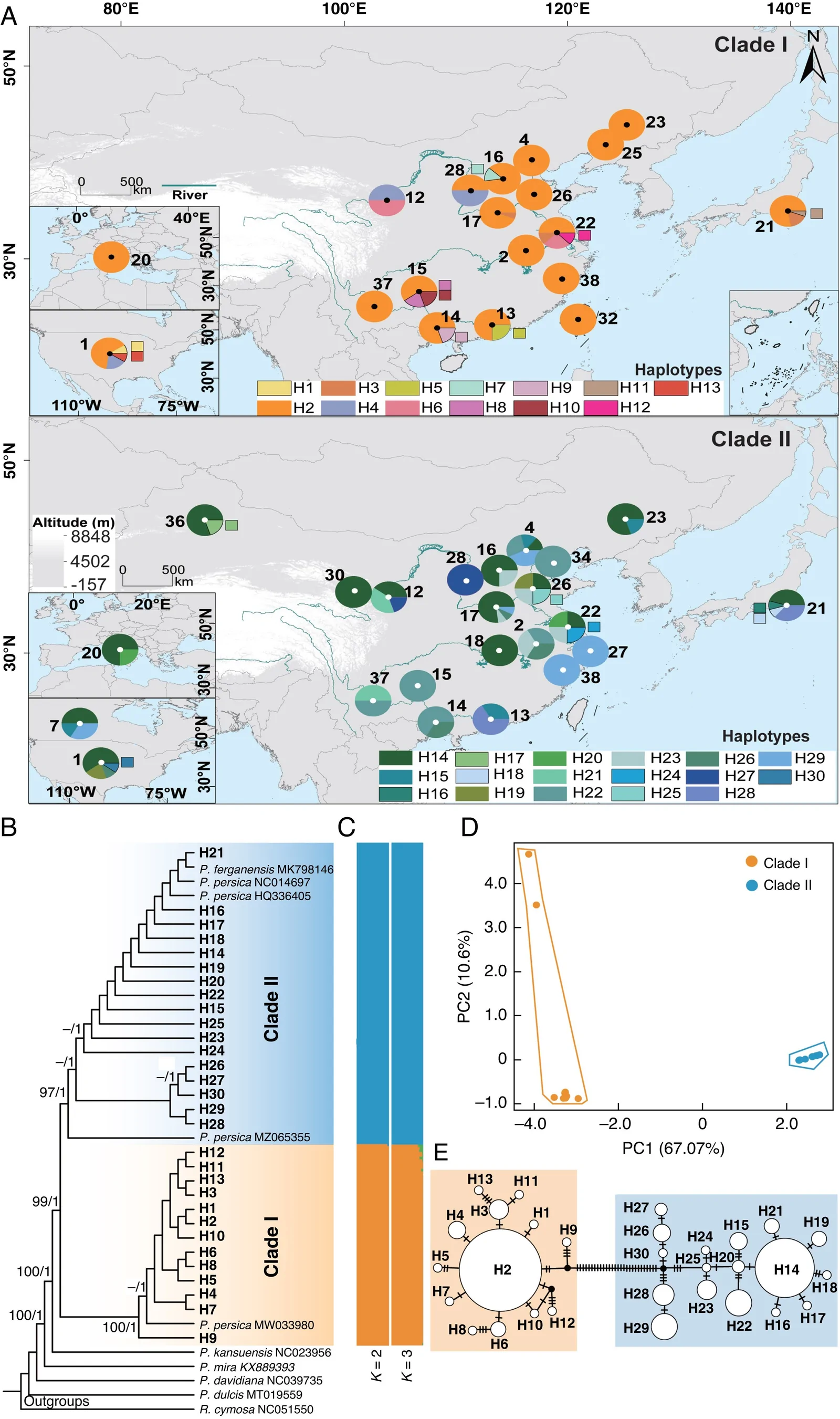
Distribution and phylogenetic relationships of cpDNA haplotypes in the landrace and wild (LW) group of peach. (A) Geographical distribution of cpDNA haplotypes in Clades I (H1–H13) and II (H14–H30), with black dots for Clade I and white dots for Clade II sampling sites (see Supplementary Data Table S1 for geographic coordinates). Pie charts show haplotype frequencies at each site, with segments representing individual haplotypes (colour-coded; see key); numbers reflect the sample size at each site. Bars adjacent to pie charts indicate the number of private haplotypes per site, with bar colours corresponding to specific private haplotypes. (B) Phylogenetic tree reconstructed using ML. Nodal support is represented by ML bootstrap values (>95 %), and given that the ML and BI trees shared identical topologies, BI posterior probabilities (>0.95) are also provided at the corresponding nodes. (C) Bar plot showing ancestry proportions from STRUCTURE analysis (*K* = 2, optimal; see the Haplotype diversity, population structure and haplotype genealogy construction subsection in the Materials and methods section) based on cpDNA SNPs, with two colours (orange: Clade I; blue: Clade II) representing distinct genetic clusters. (D) Principal Component Analysis (PCA) of cpDNA haplotypes, with PC1 and PC2 axes explaining 67.07 % and 10.6 % of variance, respectively. Points represent individual haplotypes, coloured by clade (orange: Clade I; blue: Clade II). (E) Haplotype network constructed using the TCS method for cpDNA, with circle sizes proportional to haplotype frequency (e.g. H2: 89 individuals; H14: 46 individuals). Lines connecting circles indicate mutation steps (numbers denote steps). Clades I (H1–H13, orange) and Clade II (H14–H30, blue). 28 mutations are separating the two clades. The two clades are separated by a total of 28 mutations.

**Fig. 2.**
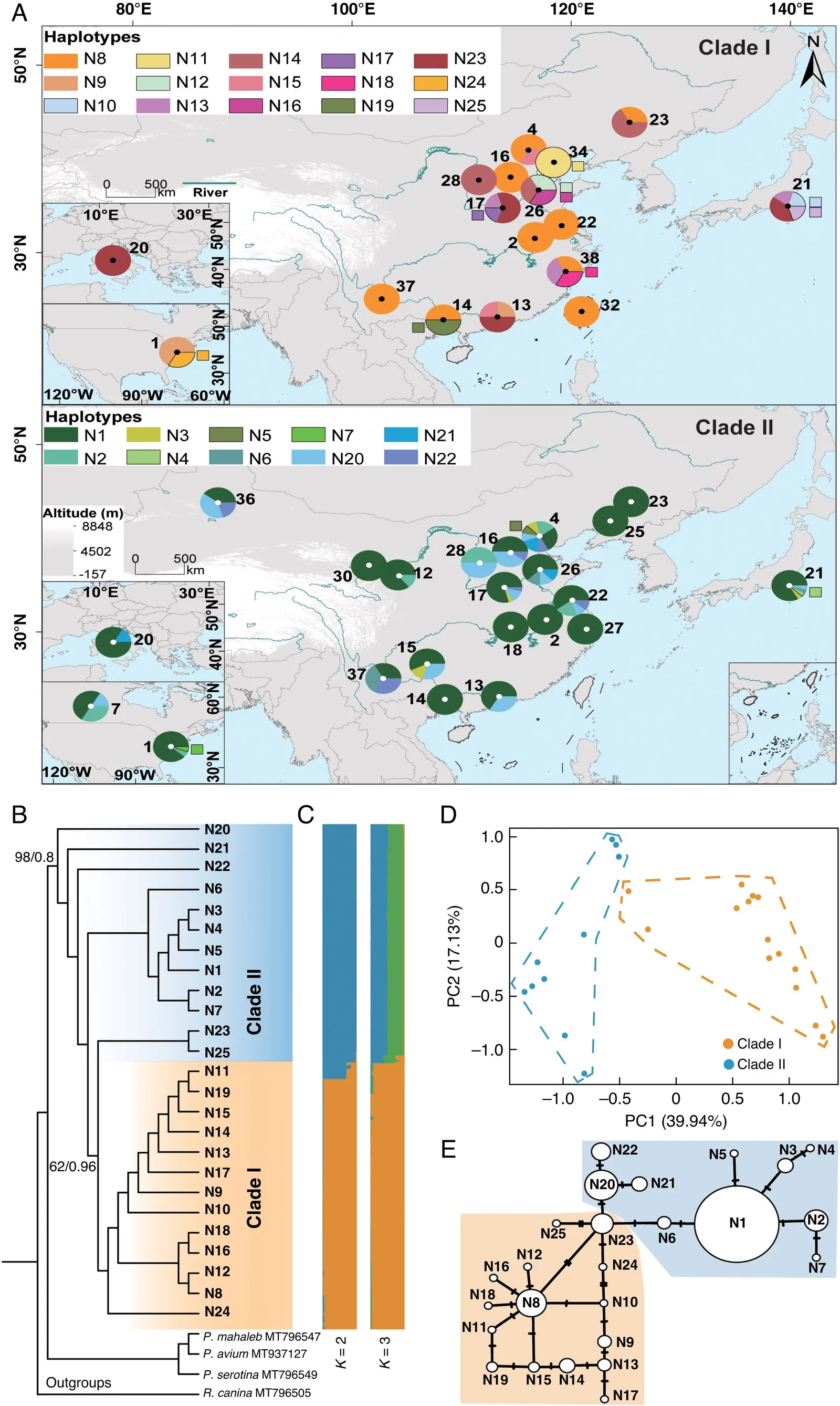
Distribution and phylogenetic relationships of nrDNA ribotypes in the landrace and wild (LW) group of peach. (A) Geographical distribution of nrDNA ribotypes in Clades I and II, with black dots for Clade I and white dots for Clade II (see Supplementary Data Table S1 for geographic coordinates). Pie charts show haplotype frequencies at each site, with segments representing individual haplotypes (colour-coded; see legend). Bars adjacent to pie charts indicate the number of private haplotypes per site, with bar colours corresponding to specific private haplotypes. (B) Phylogenetic tree of nrDNA ribotypes reconstructed using ML, including outgroups 28 Rosales species (Supplementary Data Table S2). Due to the high topological similarity between the ML and BI trees, the ML tree is presented, with nodes by ML bootstrap values (>95 %) and BI posterior probabilities (>0.6) (C) Bar plot showing ancestry proportions from STRUCTURE analysis (K = 2–3) based on nr DNA, with two colours (orange: Clade I; blue: Clade II) representing distinct genetic clusters. (D) PCA of nrDNA ribotypes, with PC1 and PC2 axes explaining 39.94 and 17.13 % of variance, respectively. Points represent individual haplotypes, coloured by clade (orange, Clade I; blue, Clade II). (E) Ribotype network constructed using the TCS method for nrDNA, with circle sizes proportional to haplotype frequency. Lines connecting circles indicate mutation steps (numbers denote steps).

Within the LW group, we identified 29 cpDNA haplotypes; Clade I consisted of 13 haplotypes (H1–H13), while Clade II consisted of 16 haplotypes (H14–H30). Within Clade I, haplotype H2 was the most frequent (89 individuals) with the widest distribution, followed by haplotype H14 in Clade II (46 individuals). The haplotype network analysis revealed that dominant haplotypes (e.g. H2) occupy central positions in the network and are surrounded by numerous low-frequency haplotypes with only one to three mutations, forming a typical star-shaped pattern for Clade I, suggesting a history of population expansion (Fig. 1E). Notably, 28 mutations separate the two clades, indicating deep divergence between maternal lineages. These findings strongly support that peach can be optimally divided into two subclades.

Several distinct haplotypes were identified as occurring in a single germplasm type or being exclusively spread across one to two areas in East Asia. For example, haplotype H11 was found only in improved accessions from southern China, while haplotype H23 was identified in northern and north-western China, and haplotype H30 was distributed in northern China in the cpDNA dataset (Fig. 1A). Similar patterns were observed in the nrDNA dataset, with a private ribotype N9, present in northern China; ribotype N29 was found in northern China (Fig. 2A).

Private haplotypes are distributed across the two clades. For cpDNA database, Clade I contained nine private haplotypes, predominantly in eastern and southern China, while Clade II harboured six private haplotypes, found in Xinjiang Autonomous Region (Fig. 1A). Additionally, the nrDNA analysis identified 25 ribotypes, including 5 widely shared and 10 private haplotypes in Clade I (N8–N19, N23–N25), and Clade II included 9 shared haplotypes and one private haplotype (N1–N7, N20–N22). Among these, ribotypes N1, N28 and N8 exhibited the widest distribution and the highest frequency (Fig. 2A).

The phylogenetic structure analysis of nrDNA sequences roughly mirrored the cpDNA results, with both datasets revealing a similar geographical distribution for the two clades, as well as areas of private haplotype aggregation (Fig. 2A). However, a notable difference was observed in the PCA results, which demonstrated the close relative positioning of the two clades. Additionally, the STRUCTURE analysis at *K* = 3 revealed two distinct components within Clade II (Fig. 2C), possibly due to the two distinct nodes within this clade (N1 and N20) (Fig. 2E).

### Haplotype distribution among different germplasm types based on the AL group

CpDNA analysis of all accessions in the AL group (714 accessions) revealed 34 cpDNA haplotypes (Supplementary Data Table S3), 30 in the LW group (182 landrace accessions and 27 wild accessions), 26 in the CL group (42 ornamental accessions and 405 improved accessions) and 7 in the NA group (58 individuals with unknown group classification, due to insufficient data). Similarly, the nrDNA analysis identified 40 ribotypes in the AL group (Supplementary Data Table S4), 25 in the LW group, 30 in the CL group and 11 in the NA group. Of the 34 cpDNA haplotypes in the AL group, 20 (58.8 %) were shared across groups. The CL group exhibited eight private haplotypes accounting for 30.8 % of its total haplotypes, while the LW group had four private haplotypes (13.3 %) (Supplementary Data Fig. S2a, b). For nrDNA, 25 out of 40 ribotypes (62.5 %) were shared among groups, with the CL group possessing 10 private haplotypes (33.3 %) and the LW group having 6 (24.0 %) (Supplementary Data Fig. S2c, d). The population structure of the AL group shows that haplotype H1 of cpDNA (belonging to Clade I) is the most widespread among cultivated peach, followed by H16 from Clade II. Similarly, nrDNA data show that ribotypes N5, N22 (Clade II) and N26 (Clade I) are the most prevalent. Most haplotypes and ribotypes were observed in at least two distinct germplasm types, and private haplotypes were distributed across various germplasm types within the AL group (Fig. 3A, B). Private haplotypes were mainly located in East Asia, with a small number distributed in other countries (America, Japan and Italy), although these non-East Asian countries are not the original source.

**Fig. 3.**
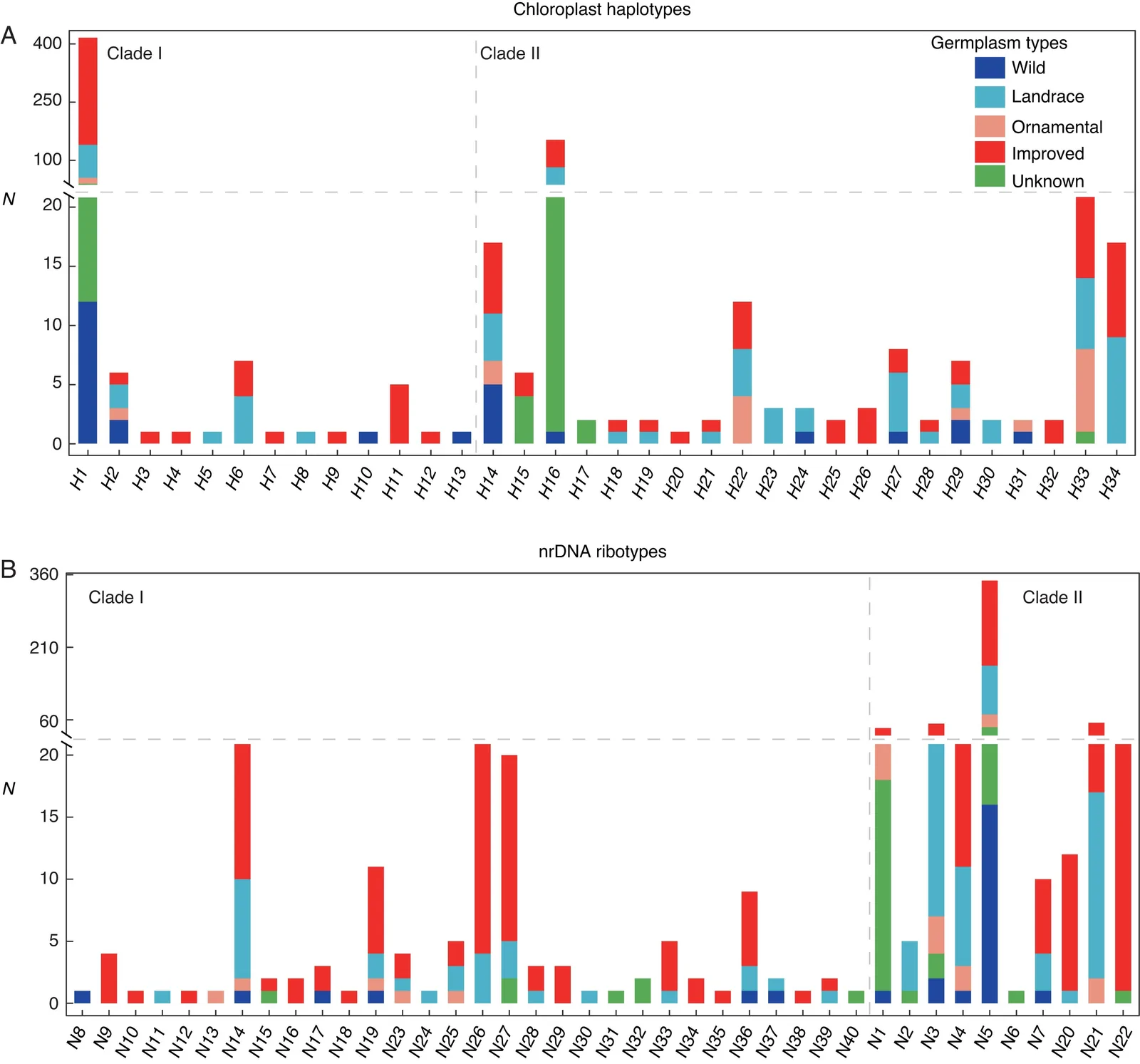
Distribution of haplotypes across different germplasm types based on cpDNA and nrDNA sequences from all individuals (AL group). (A) Bar plot showing the germplasm type composition (wild, landrace, improved, unknown accessions) of each cpDNA haplotype in the AL group, with germplasm types represented by distinct colours, and where *N* represents the number of individuals contained in each haplotype on the Y-axis (linear scale, with varying bar heights reflecting sample size. Two clades are categorized into clade I (cpDNA/nrDNA: H1–H13/N8–N19, N23–N40) and clade II (cpDNA/nrDNA: H14–H34/N1–N7, N20–N22). (B) Bar plot showing the germplasm types of composition of each nrDNA haplotype in the AL group, with colours matching those in (A).

### Genetic diversity and differentiation among populations

We evaluated genetic diversity and differentiation between the two maternal clades of peach. For the cpDNA dataset, Clade II exhibited higher haplotype diversity (*H*_d_ = 0.76) and nucleotide diversity (*π* = 3.00 × 10^−5^) than Clade I (*H*_d_ = 0.36, *π* = 1.00 × 10^−5^) (Fig. 4A). In contrast, the nrDNA dataset showed that Clade I had higher genetic diversity (*H*_d_ = 0.85, *π* = 4.00 × 10^−4^) than Clade II (*H*_d_ = 0.48, *π* = 2.20 × 10 ^−4^) (Fig. 4B).

**Fig. 4.**
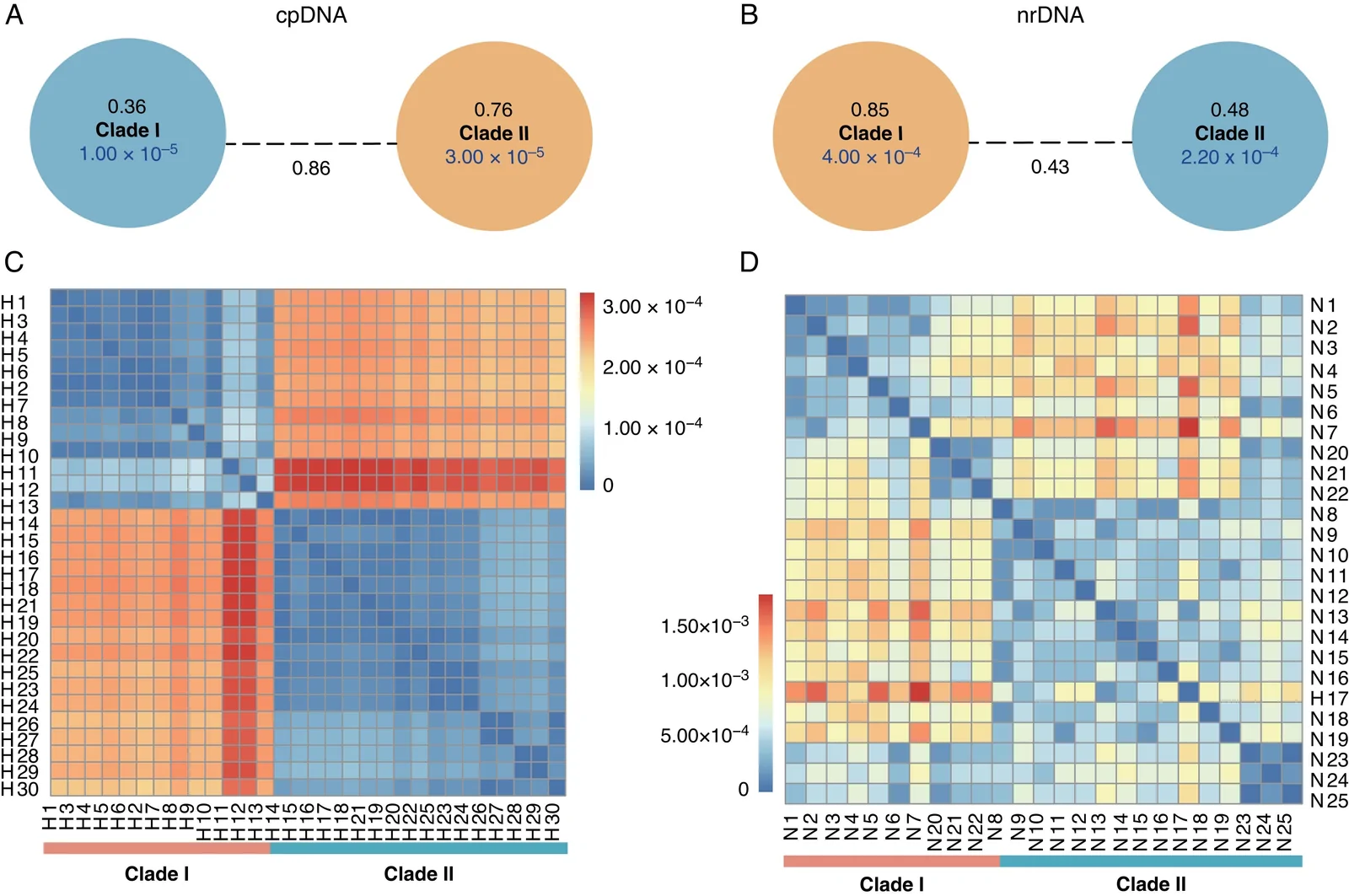
Genetic diversity and differentiation among two clades of *Prunus persica* based on cpDNA and nrDNA sequences. (A, B) Display haplotype diversity (*H*_d_) and nucleotide diversity (*π*) are shown for the two clades of peach (*P. persica*). The dashed lines connect peach Clades I and II, with annotated *F*_ST_ values indicating pairwise genetic differentiation (significant at *P* < 0.05). (C, D) Heatmaps depicting pairwise *F*_ST_ values between cpDNA haplotypes (C) and nrDNA ribotypes (D), with colour intensity indicating differentiation strength (blue, low *F*_ST_; red, high *F*_ST_; see scale bar).

Pairwise *F*_ST_ analysis indicated substantial genetic differentiation between the two clades (*F*_ST_ = 0.86, *P* < 0.05) based on cpDNA. The cpDNA *F*_ST_ heatmap further supported this pattern, showing pronounced inter-clade differentiation (mean *F*_ST_ = 0.86, range 0.80–0.92), in contrast to significantly lower within-clade differentiation (mean *F*_ST_ = 0.15, range 0.10–0.30, *P* < 0.05) (Fig. 4C). Consistently, nrDNA data showed comparable inter-clade divergence (*F*_ST_ = 0.78–0.84), most haplotypes exhibiting low within-clade differentiation, except for N20 and N23, which displayed slightly higher values (*F*_ST_ = 0.35–0.40) (Fig. 4D).

Genetic diversity indices across the different groups show that among the four germplasm types, wild accessions (*H*_d_ = 0.86, *π* = 1.30 × 10^−4^) and ornamental accessions (*H*_d_ = 0.81, *π* = 1.40 × 10^−4^) exhibit the highest genetic diversity within the cpDNA dataset, while improved accessions displayed the lowest diversity (*H*_d_ = 0.50, *π* = 1.00 × 10^−4^). Conversely, the nrDNA dataset indicated that improved accessions had the maximum haplotype diversity (*H*_d_ = 0.79) and nucleotide diversity (*π* = 5.50 × 10^−4^), suggesting their development possibly involved multiple genetic sources, while the other three germplasm types displayed similar levels of genetic diversity, with the *H*_d_ and *π* between 0.61 to 0.68 and 3.80 × 10^−4^ to 4.30 × 10^−4^, respectively. Notably, the landrace accessions exhibited the greatest nucleotide diversity (*π* = 4.30 × 10^−4^) within the nrDNA dataset.

Within China, Gansu Province (*H*_d_ = 0.91, *π* = 1.80 × 10^−4^) showed the highest haplotype and nucleotide diversity, based on the cpDNA dataset, while the Xinjiang Autonomous Region exhibited significantly lower genetic diversity (*H*_d_ = 0.40, *π* = 2.54 × 10^−6^) (Supplementary Data Fig. S5a). The nrDNA dataset revealed Beijing as the region with the highest diversity (*H*_d_ = 0.93, *π* = 5.90 × 10^−4^), whereas Gansu Province (*H*_d_ = 0.29, *π* = 5.00 × 10^−5^) and Anhui Province (*H*_d_ = 0.33, *π* = 2.4 × 10^−4^) had the lowest diversity levels (Supplementary Data Fig. S5b). Interestingly, regions situated in both the northern and southern parts of China displayed comparable genetic diversity levels. For instance, Gansu Province (*H*_d_ = 0.91, *π* = 1.30 × 10^−4^), belonging to the Yellow River region, and Jiangsu Province (*H*_d_ = 0.89, *π* = 1.40 × 10^−4^), belonging to the Yangtze River region, exhibited similar diversity in the cpDNA, while Beijing and Guangdong Province (*H*_d_ = 0.91, *π* = 5.50 × 10^−4^) (Pearl River) showed similar diversity in the nrDNA dataset (Supplementary Data Fig. S5).

### Divergence and demographic history of the maternal lineages

Divergence time estimations based on chloroplast genomes using four fossil calibration points (101–45 Ma) indicate that peach diverged from *P. kansuensis* 8.61 Ma, then Clades I and II of peach diverged from a common ancestor ∼7.21 Ma (95 % HPD 3.94–11.4 Ma**)**. The crown ages of Clade I (5.45 Ma; 95 % HPD 2.33–9.25 Ma) and Clade II (5.57 Ma; 95 % HPD 2.46–9.52 Ma) were comparable, supporting a scenario of simultaneous divergence of the two major maternal lineages within peach during the late Miocene to early Pliocene (Fig. 5B).

**Fig. 5.**
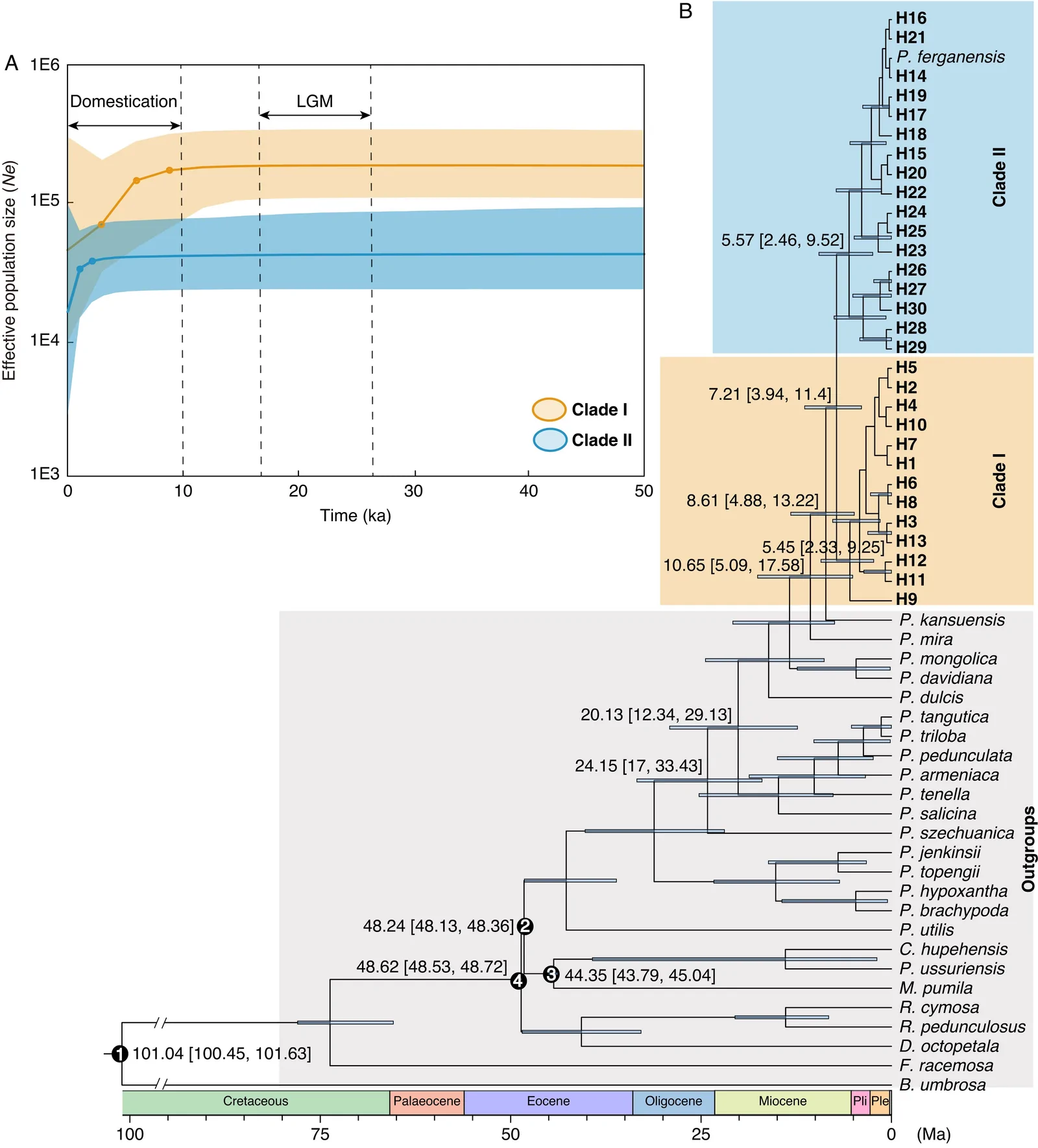
Population demography and divergence time of peach inferred from chloroplast genomic data. (A) Bayesian skyline plots at clade levels (Clade I and Clade II) of peach, with the median estimated effective population sizes represented by coloured solid lines, nesting within the 95 % highest posterior density intervals (light-shaded areas), with different colours representing different clades. (B) Divergence time estimated for peach based on the BEAST analysis, with outgroup species downloaded from NCBI (Supplementary Data Table S2). The numbers above the nodes indicate the divergence time, and the blue bars on the node represent the 95 % HPD credibility intervals. Nodes marked by black circles with numbers correspond to four fossil calibration points used. Pli refers to the Pliocene, Ple to the Pleistocene, LGM to the Last Glacial Maximum (18–26.5 ka) and Domestication refers to the period when humans began to cultivate crops (∼10 ka).

Demographic history reconstruction using Bayesian skyline plots (BSPs) based on cpDNA data further illustrated temporal changes in effective population size (*N*_e_). They revealed contrasting demographic histories for the two maternal lineages in the past. Both clades showed initial demographic stability (during the late Pleistocene), followed by population declines after ∼10 000 years ago (Fig. 5A). Clade I experienced a gradual decline in *N*_e_ beginning from 10 000 years ago, followed by a significant decline from 6000 to 3000 years ago. In contrast, Clade II remained stable and exhibited a rapid *N*_e_ decline starting around 2000 years ago, with a marked reduction in the past 1000 years (Fig. 5A).

## DISCUSSION

### Dual maternal origins and phylogeographic structure of peach

Understanding genetic structure provides remarkable insight into the evolutionary and domestication history of plants (Burke *et al.*, 2007; Alam and Purugganan, 2024; Rajora, 2024). In peach, numerous studies have employed various nuclear markers to characterize genetic structure and to explore the geographic and ecological correlates of population differentiation (Koohi Dehkordi *et al.*, 2020; Abdallah *et al.*, 2024; Demirel *et al.*, 2024; Rozanova and Vodiasova, 2025). These results have effectively partitioned peach germplasm into wild, landrace and improved accessions, and have further delineated Occidental and Oriental lineages (Cao *et al.*, 2014; Guan *et al.*, 2019; Li *et al.*, 2025; Supplementary Data Table S5). Additional population structuring has been documented across major geographic regions, namely the Yunnan–Guizhou Plateau, north-western China and north-eastern China (Li *et al.*, 2021, 2025; Supplementary Data Table S3). Despite these advances, the maternal origin and domestication history of peach remain poorly understood. Here we address this long-standing issue by integrating chloroplast (cpDNA) and nuclear ribosomal DNA (nrDNA) markers. Our analyses uncover a previously unrecognized genetic architecture comprising two distinct maternal lineages (Clade I and Clade II; Figs 1 and 2, Supplementary Data Figs S2, S3 and S4). This robust phylogeographic framework provides a coherent basis for reassessing the domestication trajectory of peach.

Although Clades I and II show considerable geographical overlap, their concentration in East Asia (Figs 1 and 2) reaffirms this region as the primary region of origin for peach (Zheng *et al.*, 2014; Li *et al.*, 2025). The uneven distribution of haplotypes across wild, landrace and improved accessions indicates that the genetic diversity of modern peaches is fundamentally rooted in these two maternal lineages. Molecular dating places the divergence between these clades at ∼8.61 Ma for the emergence of peach and ∼5.45/5.57 Ma for its subsequent diversification, substantially predating human intervention (Fig. 5B), and suggests that the two lineages were independently brought into cultivation. While additional sampling and genomic evidence are needed to corroborate this scenario, our findings collectively support a ‘dual maternal origin’ model, in which global peach germplasm derives from two ancient, independent progenitors.

### Diffuse origins of peach domestication in China

The geographic origin of cultivated plants has long been interpreted through two competing frameworks: Vavilov's ‘center of origin’ theory, which posits a primary locus of concentrated genetic diversity, and Harlan's ‘diffuse origins’ hypothesis, which allows for domestication across broad areas without a singular focus (Harlan, 1986; Vavilov and Dorofeev, 1992). Our findings, characterized by a fragmented distribution of high genetic diversity and private haplotypes across the Yellow, Yangtze and Pearl River basins (Fig. 6, Supplementary Data Fig. S5), are more consistent with the diffuse paradigm. This pattern is reinforced by the presence of ancestral haplotypes H9 in southern China and H7 in the north (Figs 1A and 2A), indicating the progenitors of domesticated peach populations were established across disparate ecological niches long before intensive human selection.

**Fig. 6.**
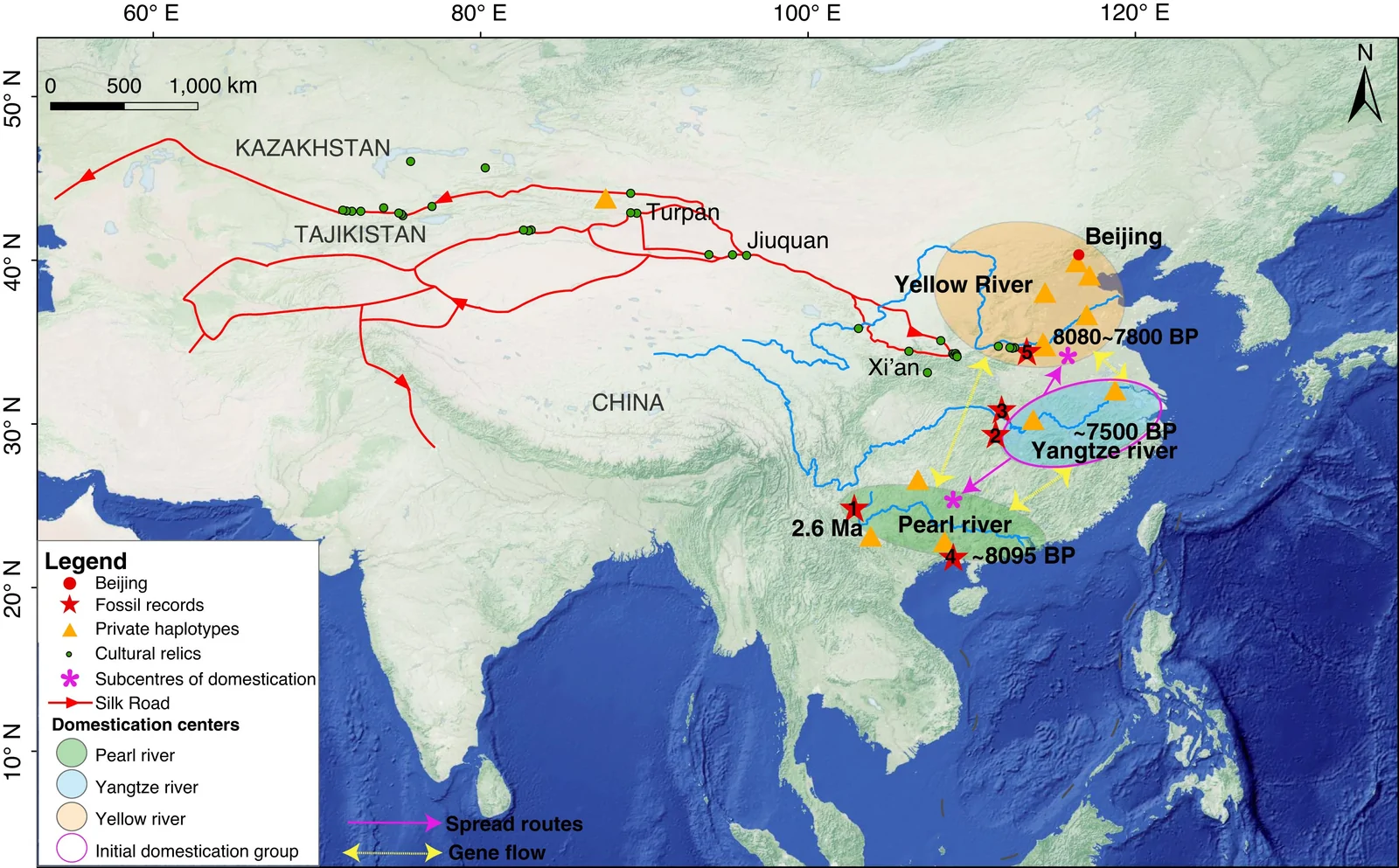
Schematic map of domestication regions and dispersal routes of peach in China. Map showing China and adjacent regions, with a scale bar indicating geographic distances. Coloured shaded areas represent the proposed domestication regions, including the middle and lower Yellow River region, the middle and lower Yangtze River region, and the Pearl River region. Orange triangles mark private cpDNA haplotypes and nrDNA ribotypes in the LW group. Red stars mark fossil and archaeological records of peach reported in previous studies (Zheng *et al.*, 2014; Su *et al.*, 2015; Yu and Fang, 1982). Green dots and red arrows trace the ancient Silk Road, which played a key role in peach dispersal. Yellow arrows indicate potential germplasm exchange among domestication regions. Following the interpretation of earlier studies (Guan *et al.*, 2019; Li *et al.*, 2025), the purple circle represents the proposed domestication origin in the Yangtze River region. Purple arrows show the likely routes of dispersal to both northern and southern regions, and purple stars indicate inferred secondary domestication regions.

Previous studies have favoured a single-centre model, identifying the middle and lower reaches of the Yangtze River as the primary region of origin for peach based on high genetic diversity and the formation of a distinct clade in this region (Guan *et al.*, 2019; Fig. 6, Supplementary Data Table S3), with subsequent dispersal giving rise to secondary regions (Li *et al.*, 2025). However, this Yangtze-centric view is difficult to reconcile with the genetic mosaic observed in our study, which shows substantial diversity and ancient haplotypes in both the Yellow River and Pearl River basins (Fig. 6, Supplementary Data Fig. S5). Moreover, this mosaic geographically coincides with the major agricultural regions of ancient China, where foundational crops such as rice (*Oryza sativa*), foxtail millet (*Setaria italica*) and various tubers were independently domesticated (Zhao, 2011; He *et al.*, 2020, 2024). Archaeological evidence further corroborates this multi-regional pattern, with peach remains unearthed in the Yellow River (Henan), the Yangtze River (Zhejiang; 8000–7000 BP) and the Pearl River basin (Guangxi; 8095 BP) (Yu and Fang, 1982; Zheng *et al.*, 2014). Collectively, these lines of evidence challenge the conventional single-centre model and point instead to a diffuse process, in which peach domestication unfolded concurrently across multiple regions in eastern China, particularly along the middle and lower reaches of the three major river systems, rather than radiating from a solitary point of origin.

This diffuse origins scenario is further reflected in the genetic diversity patterns across different germplasm types. Domestication is typically accompanied by a reduction in genetic diversity due to population bottlenecks (Allaby *et al.*, 2019; Gao *et al.*, 2019). Our data, however, reveal a more nuanced pattern: while improved accessions exhibit the lowest diversity in the uniparental cpDNA dataset (Table 1), they maintain the highest diversity in the biparental nrDNA dataset. This cytonuclear discordance suggests that maternal lineages were narrowed through selection (Birky, 1995; Wang *et al.*, 2022), whereas frequent germplasm exchange among disparate domestication regions facilitated the maintenance, or even expansion, of nuclear diversity. This is because biparentally inherited nrDNA can recover diversity through recombination, while cpDNA, which lacks recombination, has a limited capacity to restore diversity following selective sweeps (Birky, 1995; Sloan *et al.*, 2018). Such inter-regional exchange likely catalysed the emergence of the extensive germplasm type diversity observed in modern peach varieties (Fig. 3).

**Table 1.** Genetic diversity of peach across different germplasm types based on cpDNA and nrDNA datasets.

Groups	Datasets	Germplasm types/clades	*N*	Number of haplotypes	Number of private haplotypes	Haplotype diversity (*H*_d_)	Nucleotide diversity (*π*)
AL	cpDNA						
		Wild	27	13	2	0.86	1.30 × 10^−4^
		Landrace	182	30	2	0.76	1.30 × 10^−4^
		Ornamental	42	10	0	0.81	1.40 × 10^−4^
		Improved	405	25	7	0.50	1.40 × 10^−4^
		NA	58	7	4	0.64	1.20 × 10^−4^
		All	714	34	12	0.61	1.10 × 10^−4^
	nrDNA						
		Wild	27	11	2	0.66	3.60 × 10^−4^
		Landrace	182	23	5	0.68	4.30 × 10^−4^
		Ornamental	42	10	1	0.61	3.80 × 10^−4^
		Improved	405	29	8	0.79	5.50 × 10^−4^
		NA	58	11	6	0.67	6.10 × 10^−4^
		All	714	40	16	0.74	5.20 × 10^−4^
LW	cpDNA						
		Landrace + Wild	209	30	15	0.77	1.20 × 10^−4^
		Clade I	110	14	10	0.36	1.00 × 10^−5^
		Clade II	99	18	7	0.76	3.00 × 10^−5^
	nrDNA						
		Landrace + wild	209	25	12	0.67	4.20 × 10^−4^
		Clade I	45	15	9	0.85	4.00 × 10^−4^
		Clade II	164	10	3	0.48	2.20 × 10^−4^
CL	cpDNA						
		Ornamental + improved	447	26	14	0.54	1.00 × 10^−4^
	nrDNA						
		Ornamental + improved	447	30	11	0.77	5.40 × 10^−4^

AL, all accessions; LW, Landrace and wild accessions; CL, Ornamental and improved accession; NA, unknown accession.

### Human impact on peach bottlenecks and dispersal

Human domestication and cultivation of crops span at least 10 000 years, during which many plants were cultivated and domesticated (Doebley *et al.*, 2006; Zohary *et al.*, 2012; Gutaker and Purugganan, 2024). Over time, this process of human selection and dispersal resulted in genetic bottlenecks within crop populations, leading to reductions in effective population size and genetic diversity (Gupta, 2004; Abbo and Gopher, 2020; Igamberdiev, 2025). Our demographic reconstructions reveal that peach populations also experienced similar sequential founder events during dispersal of domesticates, showing two significant bottlenecks in the maternal lineages (Clade I and Clade II) (Fig. 5A). The earliest, occurring ∼6000 years ago and primarily affecting Clade I, suggests that certain wild populations underwent human selection and cultivation prior to this period. This genomic signature aligns with archaeological fossil evidence (Zheng *et al.*, 2014), which predates this genetic shift and marks the onset of intensive human-mediated evolution.

The most recent bottleneck, ∼2000 years ago, coincided with the emergence of the Silk Road, which is believed to have significantly facilitated the westward spread of peach (Li *et al.*, 2025; Fig. 5A). We hypothesize that this bottleneck arose from a series of sequential founder events accompanying dispersal along this trade route. Notably, the population decline was more pronounced in Clade II than in Clade I, suggesting that these two lineages experienced distinct selective pressures and dispersal histories. One possible explanation is that Clade II, being more geographically widespread, was subjected to more intensive and prolonged human selection, thereby accumulating a stronger genetic bottleneck signature. Alternatively, Clade II may have been preferentially selected for traits favoured during westward expansion, intensifying the bottleneck in this lineage. Together, these findings highlight the profound impact of human intervention in shaping the evolutionary trajectory of peach, where sequential founder events and trade-route-driven dispersal created distinct genetic imprints across different maternal clades (Allaby *et al.*, 2008). Similar dynamics have been documented in grapevine (*Vitis vinifera*), which experienced a prolonged domestication bottleneck and serial founder effects during its spread (Zhou *et al.*, 2017; Dong *et al.*, 2023).

### Conclusions

By integrating uniparentally inherited cpDNA and biparentally inherited nrDNA sequences, this study provides a comprehensive phylogeographic reconstruction of peach evolution. Our findings support a dual maternal origin model, demonstrating that modern cultivars derive from two distinct maternal lineages that diverged millions of years prior to human intervention. Furthermore, the spatial distribution of genetic diversity across the Yellow, Yangtze and Pearl River basins supports a diffuse origins paradigm, characterized by multiple, concurrent domestication events throughout China. Superimposed on this deep-time pattern, subsequent human-mediated dispersal along trade routes, particularly the Silk Road, further moulded the contemporary genetic structure of peach. Beyond refining the evolutionary narrative of peach germplasm, these results establish a strategic baseline for genomic-assisted breeding and provide a robust methodological framework for deciphering the complex maternal trajectories of other perennial crop species.

## Supplementary Material

mcag248_Supplementary_Data

## Data Availability

The datasets presented in this study are available in online repositories. The names of the repository/repositories and accession numbers can be found in the article/Supplementary Material. The supporting figures and tables are available at https://docs.google.com/spreadsheets/d/1NabXAnBR0gDuzr8PmhCRGw0Kjai1zb5Z/edit?usp=sharing&ouid=106574613556508739719&rtpof=true&sd=true.

## SUPPLEMENTARY DATA

Supplementary data are available at *Annals of Botany* online and consist of the following. Figure S1: geographical distribution of peach samples in this study. Figure S2: results of the STRUCTURE analysis based on two datasets from the LW group. Figure S3: phylogenetic tree constructed using ML and BI methods from chloroplast genome data based on 34 haplotypes from all 714 peach individuals (AL group), with nodes labelled by bootstrap values exceeding 95 % and posterior probabilities above 0.95. Figure S4: geographical distribution of 34 haplotypes in Clade I and Clade II of 714 peach individuals (AL group), as inferred from chloroplast genome data. Figure S5: genetic diversity across different provinces of China based on chloroplast genomic and nrDNA sequences. Table S1: list of peach samples used in this study and their haplotype selected results. Table S2: list of other Rosales species from NCBI. Table S3: Summary of genetic structure analyses of peach *(Prunus persica*) from previous studies. Table S4: GenBank accession numbers for the 34 chloroplast genome haplotypes (H1–H34) identified in *Prunus persica*. Table S5: GenBank accession numbers for the 40 nuclear ribosomal DNA (nrDNA) ribotypes (N1–N40) identified in *Prunus persica*.
