# Supplementary material for: Dual maternal origin and dispersal history of peach (Prunus persica) from China
Source: Ann Bot. 2026 Aug 5;138(5):1764–78. doi: 10.1093/aob/mcag248 (PMC13715006; doi:10.1093/aob/mcag248)
Supplement: Supplementary file 1 [file mcag248_supplementary_data.zip › Supplementary_Figures.docx]

**Annals of Botany**

Supplementary Figures for

**Dual maternal origin and diffusion history of peach (*Prunus persica*) from China**

**Figure S1.** Geographical distribution of peach samples in this study. The dots represent the sample sites.

**Figure S2.** Distribution of haplotypes across different groups and results of STRUCTURE analysis based on two datasets from LW group. **a**, Colors represent different ecotypes, with each pie chart indicating the frequency of each haplotype. **b**, mirrors this information for the nrDNA dataset, ensuring an easy comparison. Different colors of blocks represent the ecotypes**.** **c**, The Delta *K* of STRUCTURE analysis from chloroplast genome data. **d**, The Delta *K* of STRUCTURE analysis from nrDNA sequence data.

**Figure S3.** Phylogenetic tree constructed using Maximum Likelihood (ML) and Bayesian Inference (BI) methods from chloroplast genome data based on 34 haplotypes from all 714 peach individuals (AL group), with nodes labelled by bootstrap values exceeding 95% and posterior probabilities above 0.95.

**Figure S4.** Geographical distribution of 34 haplotypes in Clade I and Clade II of 714 peach individuals (AL group), as inferred from chloroplast genome data. The pie charts reflect different haplotypes, while the bars next to the pie chart indicate the type and number of private haplotypes.

**Figure S5.** Genetic diversity across provinces of China based on chloroplast genomic and nrDNA sequences. The line charts represent nucleotide diversity *(π*), while the bar charts depict haplotype diversity (*H*_d_). **a** illustrate genetic diversity based on chloroplast genomic, while **b** shows genetic diversity based on nrDNA sequences.
